# Functional Integration of Adult-Generated Neurons in Diabetic Goto-Kakizaki Rats

**DOI:** 10.3389/fnbeh.2021.734359

**Published:** 2021-10-05

**Authors:** Chelsey C. Damphousse, Jaclyn Medeiros, Diano F. Marrone

**Affiliations:** Department of Psychology, Wilfrid Laurier University, Waterloo, ON, Canada

**Keywords:** dentate gyrus, neurogenesis, spatial cognition, IEG expression, diabetes

## Abstract

Adult-born neurons in the dentate gyrus (DG) make important contributions to learning as they integrate into neuronal networks. Neurogenesis is dramatically reduced by a number of conditions associated with cognitive impairment, including type 2 diabetes mellitus (T2DM). Increasing neurogenesis may thus provide a therapeutic target for ameliorating diabetes-associated cognitive impairments, but only if new neurons remain capable of normal function. To address the capacity for adult-generated neurons to incorporate into functional circuits in the hyperglycemic DG, we measured Egr1 expression in granule cells (GCs), BrdU labeled four weeks prior, in Goto-Kakizaki (GK) rats, an established model of T2DM, and age-matched Wistars. The results indicate that while fewer GCs are generated in the DG of GK rats, GCs that survive readily express Egr1 in response to spatial information. These data demonstrate that adult-generated GCs in the hyperglycemic DG remain functionally competent and support neurogenesis as a viable therapeutic target.

## Introduction

Type 2 diabetes mellitus (T2DM) has long been associated with cognitive impairment and an increased risk of dementia. In fact, this association appeared in literature over 70 years ago with the introduction of the term diabetic encephalopathy to describe CNS-related complications of diabetes (Dejong, 1950). As our understanding has progressed, these deficits are more commonly referred to as diabetes-associated cognitive decline (DACD; Mijnhout et al., 2006). Despite a long history, the mechanisms by which T2DM undermines defined neural circuits that support cognitive function remains unknown. Clarifying the neuronal changes that drive DACD may be aided through the use of animal models such as the Goto-Kakizaki (GK) rat (Goto et al., 1975; Kimura et al., 1982), a strain selectively bred for insulin resistance and persistent hyperglycemia in the absence of obesity. Importantly, these animals display cognitive deficits (e.g., Matsunaga et al., 2016; Li et al., 2017; Tian et al., 2018; Yang et al., 2018) comparable to those exhibited by humans with T2DM (e.g., Biessels et al., 2006; Lu et al., 2009; Cukierman-Yaffe, 2014; Palta et al., 2014; Moheet et al., 2015).

Among neuronal changes described in GK rats thought to contribute to DACD is an accelerated granule cell (GC) turnover resulting in decreased survival of adult-generated granule cells (agGCs) both in the hippocampus (Lang et al., 2009; Beauquis et al., 2010) and olfactory bulb (Lietzau et al., 2018). In addition to looking for changes in the proliferation, differentiation, and survival of agGCs, it is important to assess the capacity of these cells to incorporate into functional neuronal networks that support cognition. The participation of agGCs in networks can be assessed through the expression of several activity-dependent gene products (Jessberger and Kempermann, 2003; Ramirez-Amaya et al., 2006; Kee et al., 2007; Alme et al., 2010; Stone et al., 2011), including early growth response 1 (Egr1; Tashiro et al., 2007). The present study assays the capacity of agGCs to participate in behaviorally-induced Egr1 in response to spatial information. Assessing the functional competency of these GCs is critical because it has direct implications for the treatment of DACD—if agGCs cannot integrate into neuronal networks then neurogenesis is not a viable therapeutic target and up-regulating neurogenesis may actually worsen DACD.

## Materials and Methods

All procedures were approved by the Wilfrid Laurier University Animal Care Committee, in compliance with the standards of the Canadian Council on Animal Care. Note that all reagents are from Millipore Sigma Canada (Oakville, ON) unless otherwise specified.

### Subjects

The current study includes 16 male Wistar rats (Charles River, St. Constance, QC) and 16 male Goto-Kakizaki rats (bred at Wilfrid Laurier University from stock originally obtained from Charles River). Wistar rats were individually housed upon arrival, and GK rats were moved from group housing to individual cages. All rats were 6–8 months old at the beginning of the experiment, and were given one week to acclimate to individual housing before any other procedures were conducted. All animals received an inverted 12 h light cycle and maintained with water and food *ad libitum*.

### Morris Water Maze

Spatial memory was tested with the Morris watermaze as previously described (Marrone et al., 2012; Gheidi et al., 2020). Briefly, each rat received 6 trials per day in a 1.8 m diameter pool, at a maximum of 60 s per trial, for 4 consecutive days. The platform was hidden 2 cm below the surface of water made opaque using tempera paint. At the end of day 4, a probe trial was conducted in which the platform was removed to assess the proximity of the swim path to the target location. A final test day, consisting of 12 trials in which the platform was visible, assessed sensorimotor function.

### BrdU Administration

Following water maze testing, animals received daily injections of BrdU (50 mg/kg i.p.) for 5 days. Following the injections, rats remained undisturbed in their home cages for 4 weeks.

### Spatial Exploration

Groups of 8 GK and 8 Wistar rats underwent 5 min of spatial exploration, as described previously (Ramírez-Amaya et al., 2005; Marrone et al., 2012). The exploration environment was a 70 × 70 cm open box with 20-cm-high walls, partitioned into nine grids. Each rat was moved to the center of a different grid every 15 s such that each grid was visited two or three times during the 5 min session. Rats were then immediately placed back in their home cage. Another 8 GK and 8 Wistar rats remained in their home cages as control animals.

### Tissue Preparation

Thirty minutes after the exploration session, animals were decapitated and the brains were extracted and frozen in 2-methylbutane immersed in a secondary container holding a slurry of dry ice and ethanol. Brain hemisections containing the right dorsal hippocampus from 2 brains from each group were molded into blocks with frozen section embedding media (VWR Canada, Mississauga, ON). The 4 blocks were then cryosectioned into 20 μm coronal sections, thaw-mounted on Superfrost-plus slides (VWR), dried, and stored at −70°C.

### Immunohistochemistry

One in every 10 slides from each block was selected from the dorsal hippocampus for immunohistochemistry (IHC) using previously described protocols (Ramirez-Amaya et al., 2006; Marrone et al., 2012; Meconi et al., 2015). Slides were fixed in 2% formaldehyde for 5 min, permeabilized with acetone/methanol (1:1 v/v) at 4°C for 5 min, washed in TBS, and then quenched with 1% H_2_O_2_ (in TBS) for 15 min. After blocking with Superglo blocking buffer for 30 mins (Fluorescent Solutions, Augusta, GA), the slides were incubated (4°C overnight) with anti-NeuN-HRP (1:2,000) and labeled with Superglo blue for 30 mins (Fluorescent Solutions, Augusta, GA). Slides were then incubated (4°C for 2 h) in polyclonal rabbit anti-Egr1-HRP (1:500, Santa Cruz Biotechnology, Dallas, TX), followed by Superglo red for 30 mins (1:50, Fluorescent Solutions). The DNA was then denatured with a 50% formamide/2 × SSC (1:1 v/v, Sigma) at 65°C for 2 h, incubated in 2N HCl at 37°C for 30 min, and washed in 0.1 M borate buffer (pH 8.5, Sigma) for 10 min. before incubation (4°C for 24 h) with mouse anti-BrdU-HRP (1:100, Roche) followed by Superglo green (Fluorescent Solutions). If imaging was not possible from any processed tissue (e.g., because a section was torn during sectioning), adjacent slides were selected for further processing.

### Confocal Imaging and Analysis

Imaging and analysis was conducted as described previously (Ramirez-Amaya et al., 2006; Marrone et al., 2012; Satvat et al., 2012; Meconi et al., 2015). Modifications are described below. Analysis was restricted to the suprapyramidal blade (also referred to as the dorsal blade) of the DG, as the key observation here is Egr1 expression in both developmentally- and adult-generated GCs, and this region shows most of the behaviorally-driven IEG expression in the DG in response to spatial processing (Chawla et al., 2005; Ramirez-Amaya et al., 2006; Marrone et al., 2012; Meconi et al., 2015). Images were obtained with an Olympus FV1000 (Olympus Canada, Mississauga, ON) confocal microscope with using a 40× objective. Image stacks were collected from the whole thickness of the tissue (20 μm), over the entire DG for each animal. The imaging parameters were set using a Wistar caged control on a given slide, and all images in that slide were taken with the same parameters. At least 10 whole DG regions, taken from selected sections and ranging from ~4–5.5 mm from the interaural plane (Paxinos and Watson, 2006), were imaged for each animal.

**Figure 1:**
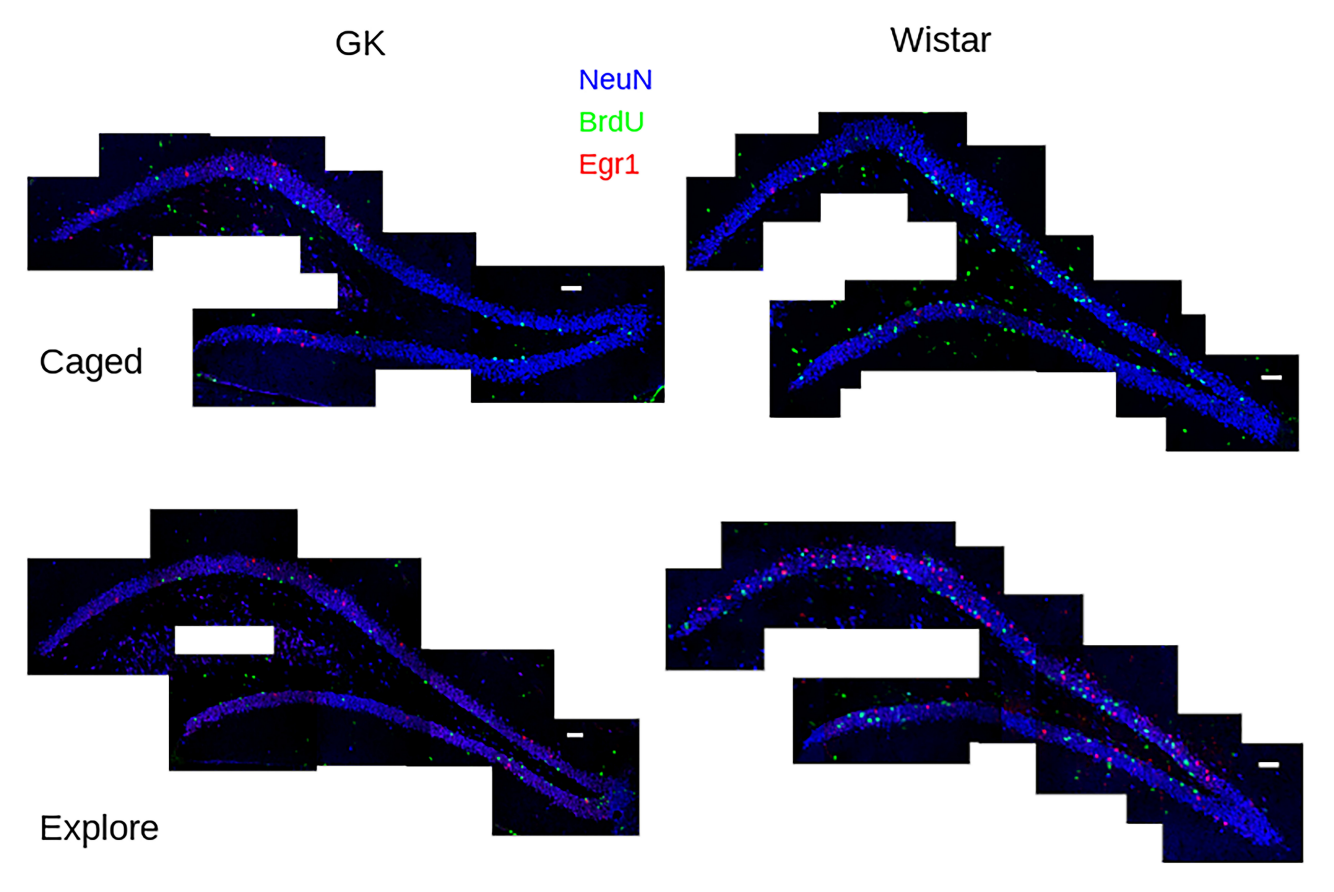
Task-dependent *Egr1* transcription. Sample tile-scans (scale bar = 100 μm) show Egr1 (red) BrdU (green) and NeuN (blue) in the dorsal dentate gyrus from each group. Fewer BrdU+ cells can be seen in GK rats (left) compared to Wistars (right) in both groups. These images show that caged control (caged, above) rats of both strains show few Egr1+ GCs. A robust up-regulation of *Egr1* can be seen, largely in the suprapyramidal blade, in both strains of rats that engaged environmental exploration (Explore, bottom), although more robust Egr1 expression occurs in Wistars.

Each DG was reconstructed with ImageJ (NIH) using the middle plane from each stack, resulting in a 2D image (Figure 1). This image then was used as the reference image, and the 40× image stack was used to identify the BrdU+/NeuN+ and Egr1+ cells. Each BrdU+ cell co-localizing with NeuN was considered a new GC, and the Egr1+ cells were considered the activated neurons. Each NeuN+ cell that was classified as BrdU+, Egr1+, or both was annotated in the reference image and verified in high-magnification confocal stacks. After all image stacks were collected and all positive cells in the suprapyramidal blade were classified, the volume of the suprapyramidal granular cell layer was calculated in the reference image, from the genu to the most dorsal tip of the granule cell layer.

### Statistics

Spatial learning trials and visual trials in the Morris water maze were analyzed with repeated measures analysis of variance (ANOVA), using training day as the repeated factor. Probe trial performance was assessed using paired-sample *t*-tests, pairing for each animal the time spent in the quadrant of the maze that previously held the platform with the opposite quadrant. Analysis of Egr1 and BrdU expression was conducted using a 2 × 2 ANOVA with strain (i.e., Wistar vs. GK) and behavioral group (i.e., exploration vs. caged controls) as factors. Egr1 expression was also analyzed independently in BrdU+ and BrdU-labeled GCs only in animals that explored a novel environment using a one-way ANOVA. All *post hoc* tests were conducted using Tukey's honest significant difference. All contrasts were calculated using JASP 0.14.3 (JASP Team, 2021).

## Results

### Goto-Kakizaki Rats Have Impaired Spatial Reference Memory

**Figure 2:**
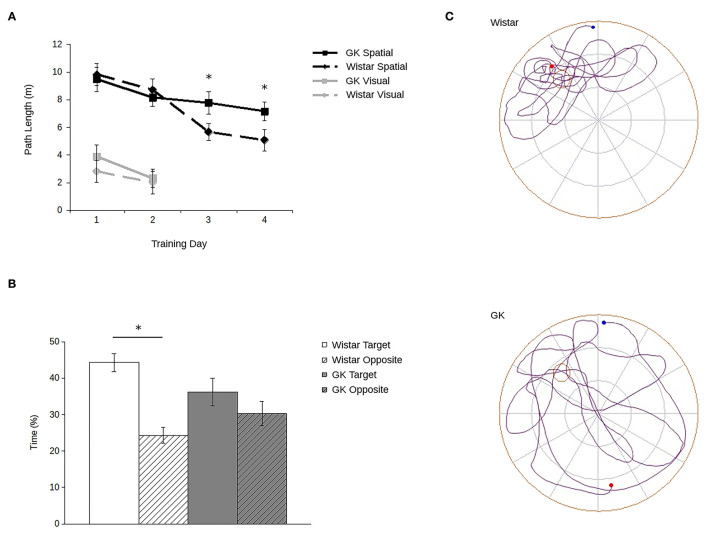
Goto-Kakizaki (GK) rats show impaired spatial reference memory in the Morris water maze. Path lengths **(A)** show that GK rats (square) swam longer paths to reach the hidden platform (black lines) than did the age-matched Wistar rats (diamond). When the platform was visible (gray lines), GKs and Wistars had comparable path lengths. During probe trials **(B)** with the platform removed, Wistars (white), but not GKs (gray) spent more time in the maze quadrant that previously contained the platform (blank) compared to the opposite quadrant (hatched). Representative path lengths **(C)** during the probe trial confirm that during probe trials, Wistar rats (above) swim from their start point (blue) to the platform's quadrant and swim a more confined pattern with an end point (red) adjacent to the platform's previous location. GK rats (below) swim through a greater proportion of the maze and at a greater distance from the platform's previous location (all data are mean ± SEM; ^*^p < 0.05, GK vs. Wistar).

Consistent with previous data (e.g., Li et al., 2017; Tian et al., 2018; Yang et al., 2018; Ke et al., 2020), GK rats showed robust spatial memory deficits in the Morris water maze (Figure 2). Importantly, they showed no deficit in their ability to locate a visible platform.

Although all rats showed improvement over trials (main effect of training day: *F*_3,90_ = 5.71, *p* = 0.001), GK rats swam longer paths to the platform relative to Wistars (Figure 2A). Although no main effect of strain was observed (*F*_1,30_ = 1.16, *p* = 0.29), a significant strain by training day interaction (*F*_1,16_ = 4.53, *p* = 0.007) shows that strain-related differences in spatial learning emerged larger over the course of training. *Post hoc* analyses confirmed that differences in path lengths between GK and Wistar rats grew larger as training time progressed, reaching significance on days 3 (*p* = 0.02) and 4 (*p* = 0.003). Similarly, during probe trials (Figures 2B,C) Wistar rats spent significantly more time in the target quadrant of the pool that previously held the escape platform compared to the opposite platform (*t*_15_ = 8.82, *p* < 0.001). In contrast, GK rats did not (*t*_15_ = 1.54, *p* = 0.07).

However, when the platform was made visible (Figure 2A, inset), no significant difference was observed between the path lengths of GKs and Wistars (main effect of strain: *F*_1,30_ = 3.19, *p* = 0.09), and all rats improved across the 2 days of training (main effect of day: *F*_1,30_ = 58.44, *p* < 0.001). A significant interaction was observed between strain and training day (*F*_1,30_ = 6.32, *p* = 0.02) was observed. *Post-hoc* tests showed that while GK rats took longer path lengths than Wistars on day 1 (*p* = 0.04), by day 2 no significant difference was apparent (*p* = 0.65). Thus, the impairments of GK rats cannot be accounted for by changes in visual acuity or motor abilities.

### Fewer Adult-Born Granule Cells Survive 4 Weeks in Goto-Kakizaki Rats

**Figure 3:**
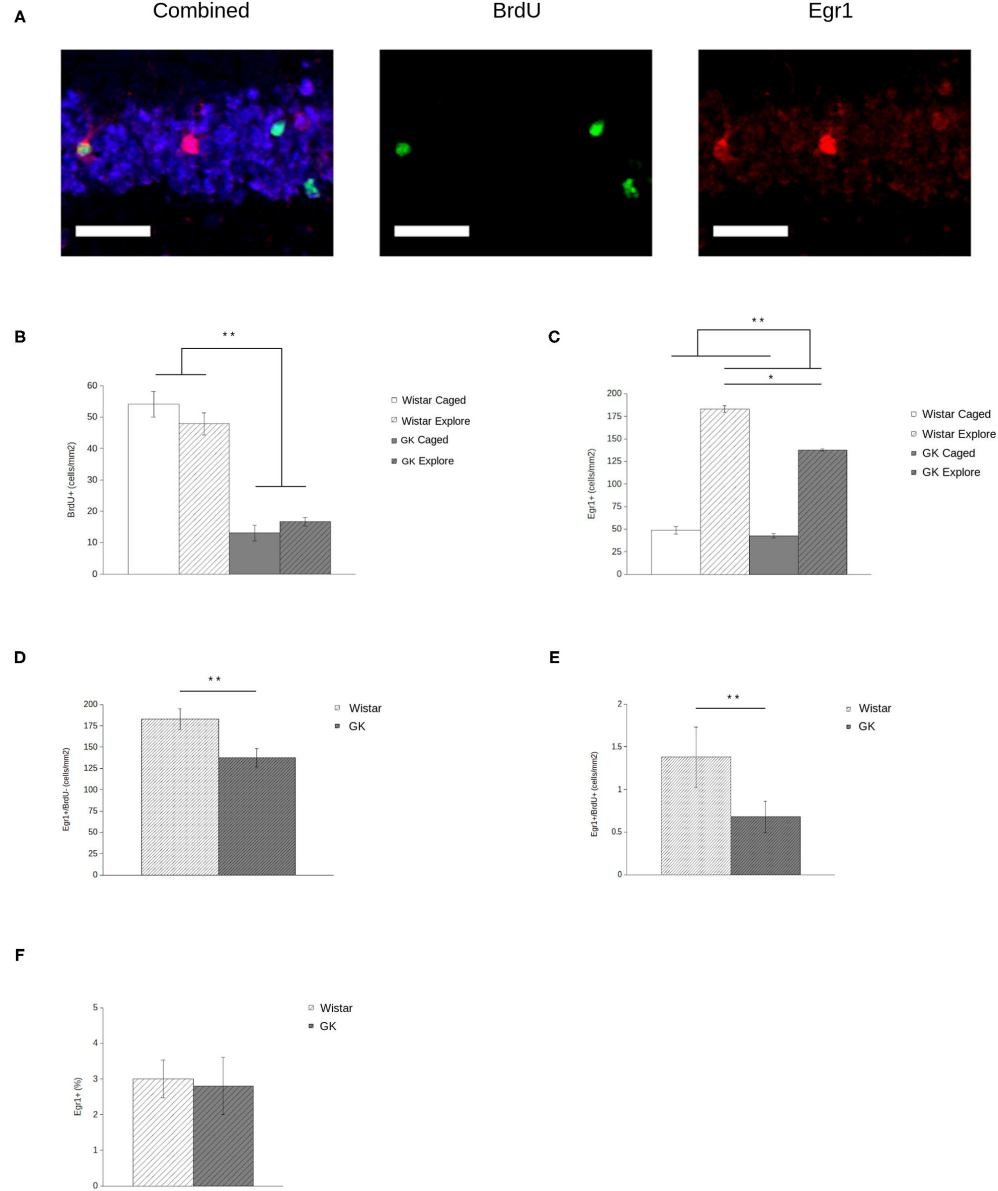
Egr1 expression in adult-generated granule cells (agGCs) is preserved in Goto-Kakizaki (GK) rats. Sample confocal projection images (**A**, scale bar = 50 μm) show GCs labeled with NeuN (blue, left), BrdU (green, middle), and Egr1 (red, right) portraying examples of cells labeled with each marker, including a BrdU+/Egr1+ neuron. Quantitative data show that **(B)** fewer BrdU+ cells were observed in GKs (gray) relative to Wistars (white) with no difference found between animals that explored a novel environment (hatched) relative to caged controls (solid). In addition **(C)**, fewer GCs express Egr1 in GKs relative to Wistars, although exploring animals show more Egr1+ GCs than caged controls. When analyzing newborn BrdU+ **(D)** and older BrdU- **(E)** GCs by volume, both show a significant decrease in GKs relative to Wistars. However, if Egr1 expression in agGCs is quantified as a proportion of all BrdU+ cells **(F)**, no difference is seen, indicating that agGCs in both strains have an equal probability of being recruited to be active during a single discrete experience (All data are mean ± SEM; ^*^p < 0.05, ^**^p < 0.001, Wistars vs. GKs).

BrdU expression (Figure 3A, middle) was significantly lower in GKs (*F*_1,28_ = 181.45, *p* < 0.001) relative to Wistars (Figure 3B), comparable to previous reports (e.g., Lang et al., 2009). In addition, exploration did not influence the number of agGCs (*F*_1,28_ = 0.11, *p* = 0.74) as expected, since BrdU injections occurred 4 weeks before exploration, long after the period in which exercise and/or spatial experience influences agGCs (e.g., Gould et al., 1999; van Praag et al., 1999; Ambrogini et al., 2000).

### Less Behaviorally-Driven Egr1 in GK Rats

Analysis of Egr1 expression (Figure 3A, right) shows that both GK and Wistar rat respond to exploration with a robust increase in the number of GCs expressing Egr1 (Figure 3C) relative to caged controls (*F*_1,28_ = 129.86, *p* < 0.001), consistent with previous results using this paradigm (e.g., Marrone et al., 2012). In GKs, behaviorally-induced Egr1 expression is significantly blunted relative to caged controls (*F*_1,28_ = 6.52, *p* = 0.02).

### Behaviorally-Induced Egr1 Is Spared in 4-Week-Old Neurons in GK Rats

When analyzing newborn (i.e., Brdu+) and older (i.e., BrdU-) GCs in exploring rats by volume, both older (*F*_1,14_ = 6.67, *p* = 0.02; Figure 3D) and newborn (*F*_1,14_ = 6.40, *p* = 0.03; Figure 3E) GCs show a significant decrease in Egr1 expression. Although this volumetric assessment provides an estimate of how many agGCs are recruited to represent a discrete experience, it does not provide an accurate assessment of their functional capabilities. Even if agGCs are functionally normal, counts of Egr1+ agGCs in GKs will necessarily be lower because there is a much smaller pool of agGCs to recruit from. By instead assessing Egr1 expression as a proportion of all BrdU+ cells, we estimate the probability that an individual agGC is recruited to be active during a single discrete experience. Using this measure (Figure 3F), no significant difference was found between strains (*F*_1,14_ = 0.03, *p* = 0.86).

## Discussion

Consistent with previous data, the current study shows that GK rats display a deficit in spatial memory that is correlated with decreased survival of agGCs. These observations are consistent with the considerable literature showing that adult-generated neurons make a critical contribution to learning by reducing memory interference and increasing cognitive flexibility (reviewed by Anacker and Hen, 2017; Miller and Sahay, 2019). The deficits seen in GK rats are also correlated with a decrease in Egr1 expression. In addition, the current observations differentiate between older GCs that show a loss of function, and agGCs that are reduced in number, but when this reduction is controlled for, are viable. It should be noted that the accuracy of comparisons of Egr1 expression in older GCs depends, in part, on the number of older GCs remaining comparable between GK and Wistar rats. Although assumption-free stereology has yet to be conducted in the DG, there are reports of degeneration in other brain regions (e.g., Lietzau et al., 2016). Despite this possibility, it is clear that the role of older GCs in information processing has been weakened, underscoring the more viable agGCs as a target for treatment.

These data provide an important demonstration that agGCs respond to spatial information despite continued hyperglycemia. Understanding how agGCs develop and process information under these conditions is vital considering that diabetic therapies such as metformin increase neurogenesis (Yoo et al., 2011; Wang et al., 2012; Ahmed et al., 2017; Tanokashira et al., 2018) but do not reverse hyperglycemia (Tanokashira et al., 2018). The notion that increasing neurogenesis without reversing hyperglycemia is sufficient to improve DACD is consistent with observations of improved cognition in diabetic individuals undergoing drug treatment relative to untreated individuals (Sato et al., 2011; Ng et al., 2014).

Although these data hold promise for neurogenesis as therapeutic target, many questions remain unanswered. Although agGCs are clearly capable of responding to spatial information, concluding that they function properly requires further investigation of the activity of agGCs over time and under varying conditions. Differences in the activity of agGCs in GKs and Wistars may emerge once age comparisons are made examining the responses of these cells over multiple events or contexts. Collecting these data is especially important considering that the stability of activity patterns across multiple behavioral episodes is predictive of memory function in individual GCs (Jaeger et al., 2018) as well as memory abilities of individual animals (Marrone et al., 2011). Moreover, chronic hyperglycemia has widespread effects on hippocampus beyond a reduction in neurogenesis that may affect information processing (reviewed by Biessels et al., 2002; Garcia-Serrano and Duarte, 2020). Even if we limit consideration to only the DG, information processing is compromised largely in developmentally-generated cells. Increasing neurogenesis may help compensate for these changes, but this does not address the potential mechanisms by which hyperglycemia is impairing (and potentially destroying) older GCs. This remains an important question as older GCs play a complementary role in memory function (e.g., Nakashiba et al., 2012; Tronel et al., 2015).

Despite these open questions, the observation that viable agGCs are generated in the hyperglycemic hippocampus satisfies an important requirement for therapies that propose to stimulate neurogenesis as a means to rescue DACD.

## Data Availability Statement

The raw data supporting the conclusions of this article will be made available by the authors, without undue reservation.

## Ethics Statement

The animal study was reviewed and approved by Wilfrid Laurier University Animal Care Committee.

## Author Contributions

CD and JM handled animals, prepared tissue, and analyzed images. CD and DM imaged tissue. CD and DM wrote the manuscript. All authors contributed to the article and approved the submitted version.

## Funding

This work was supported by Mental Health Research Canada.

## Conflict of Interest

The authors declare that the research was conducted in the absence of any commercial or financial relationships that could be construed as a potential conflict of interest.

## Publisher's Note

All claims expressed in this article are solely those of the authors and do not necessarily represent those of their affiliated organizations, or those of the publisher, the editors and the reviewers. Any product that may be evaluated in this article, or claim that may be made by its manufacturer, is not guaranteed or endorsed by the publisher.
